# Optimization and Kinetic Evaluation for Glycolytic Depolymerization of Post-Consumer PET Waste with Sodium Methoxide

**DOI:** 10.3390/polym15030687

**Published:** 2023-01-29

**Authors:** Saqib Javed, Jonas Fisse, Dieter Vogt

**Affiliations:** 1 Laboratory of Industrial Chemistry, Department of Biochemical and Chemical Engineering, TU Dortmund University, Emil-Figge-Straße 66, 44227 Dortmund, Germany; 2 Department of Chemical, Polymer, and Composite Materials Engineering, University of Engineering and Technology (UET), Lahore 39161, Pakistan

**Keywords:** chemical recycling, PET waste, sodium methoxide, response surface methodology, thermodynamic and kinetic evaluation, depolymerization reaction

## Abstract

Glycolysis of post-consumer polyethylene terephthalate (PET) waste is a promising chemical recycling technique, back to the monomer, bis(2-hydroxyethyl) terephthalate (BHET). This work presents sodium methoxide (MeONa) as a low-cost catalyst for this purpose. BHET product was confirmed by gas chromatography-mass spectrometry (GCMS), Nuclear Magnetic Resonance (NMR) Spectroscopy, melting point, and Differential Scanning Calorimetry (DSC). It was shown, not surprisingly, that PET conversion increases with the glycolysis temperature. At a fixed temperature of 190 °C, the response surface methodology (RSM) based on the Box-Behnken design was applied. Four independent factors, namely the molar ratio of PET: MeONa (50–150), the molar ratio of ethylene glycol to PET (EG: PET) (3–7), the reaction time (2–6 h), and the particle size (0.25–1 mm) were studied. Based on the experimental results, regression models as a function of significant process factors were obtained and evaluated by analysis of variance (ANOVA), to predict the depolymerization performance of MeONa in terms of PET conversion. Coefficient of determination, R^2^ of 95% indicated the adequacy for predicted model. Afterward, the regression model was validated and optimized within the design space with a prediction of 87% PET conversion at the optimum conditions demonstrating a deviation of less than 5% from predicted response. A van ‘t Hoff plot confirmed the endothermic nature of the depolymerization reaction. The ceiling temperature (*T_C_* = 160 °C) was calculated from Gibbs’ free energy. A kinetic study for the depolymerization reaction was performed and the activation energy for MeONa was estimated from the Arrhenius plot (*E_A_* = 130 kJ/mol). The catalytic depolymerization efficiency of MeONa was compared under similar conditions with widely studied zinc acetate and cobalt acetate. This study shows that MeONa’s performance, as a glycolysis catalyst is promising; in addition, it is much cheaper and environmentally more benign than heavy metal salts. These findings make a valuable contribution towards the chemical recycling of post-consumer PET waste to meet future recycling demands of a circular economy.

## 1. Introduction

In total, more than 8.3 billion tons of plastic have been produced since the early 1950s and approximately 60% ended up in landfills [1,2]. The largest groups of plastics include polyethylene (36%), polypropylene (21%), polyvinylchloride (12%), poly(ethyleneterephthalate) (PET), polyurethane, and polystyrene (<10% each) [3]. PET is the fourth most-produced thermoplastic with a production of more than 73 million tons in 2020 [4]. For the reason of being lightweight, transparent, and virtually indestructible, PET has a huge stake in drinking water and soft drink bottles [5] that have resulted in the production of 583.3 billion PET bottles in 2021 [6] PET is not directly toxic material for the environment but PET bottles take around 450 years to degrade, resulting in the generation of billions of disposable drinking bottles as post-consumer PET (POSTC-PET) waste [7]. Furthermore, PET is a petroleum-derived material, its recycling should be implemented for environmental sustainability and it can provide raw material to many industries, contributing to the conservation of high-cost petrochemical raw materials and energy.

**Scheme 1:**
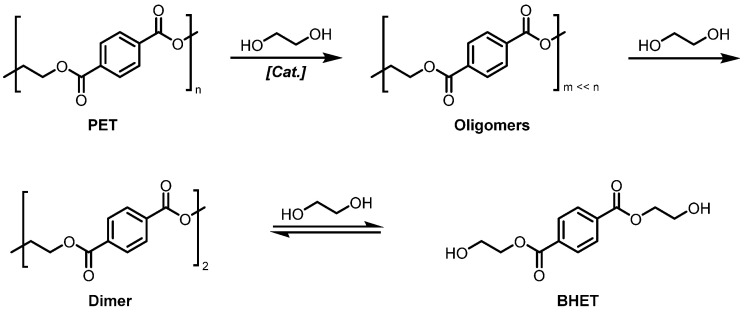
Depolymerization reaction of PET with ethylene glycol into BHET.

PET synthesis today mostly involves the esterification of terephthalic acid (TPA) with ethylene glycol (EG) to bis(2-hydroxyethyl terephthalate) (BHET), which is then subjected to a polycondensation reaction [8]. Chemical recycling is the depolymerization of PET into its monomers (TPA or BHET) or partial depolymerization into oligomers. This is achieved through various solvent-assisted techniques, including methanolysis, hydrolysis, aminolysis, ammonolysis, and glycolysis. A detailed comparison of all these chemical recycling techniques can be found elsewhere [9]. Nevertheless, most of these techniques require severe reaction conditions, such as prolonged reaction times, high temperatures and pressures, or even the presence of large amounts of concentrated acids or bases [10,11]. PET glycolysis, as shown in Scheme 1, is the molecular degradation of PET polymer by glycols, typically ethylene glycol (EG), to give bis(2-hydroxyethyl) terephthalate (BHET) [12]. PET glycolysis is the most promising method within the previously described approaches owing to the low cost, mildest operating conditions, and low volatility of solvents.

Glycolysis of POSTC-PET waste is carried out in the presence of a transesterification catalyst. Baliga et al. investigated the efficiency of metal acetate catalysts (Zn, Mn, Co, and Pb) in excess ethylene glycol (molar ratio of EG to PET = 4) at 190 °C for depolymerization of post-consumer PET waste and found that zinc acetate performed best in terms of BHET formation [13]. Afterward, Ghaemy and Mossaddegh applied similar metal acetate catalysts for the depolymerization of PET fiber waste using a molar ratio of EG to PET = 8.5 at 198 °C and came up with an interpretation that depolymerization activity with 8 h reaction time follows the order of Zn^2^ > Mn^2^ > Co^2^ > Pb^2^ [14]. Besides homogeneous metal salt-based catalysts, now heterogeneous catalysts are also widely used for PET glycolysis. Fehér et al. demonstrated that organocatalyst-modified silica gels are efficient for PET glycolysis achieving a non-isolated BHET yield of 88.5% under optimum conditions (190 °C, 1.7 h,) by handling only 384 mg of PET substrate in a reaction vial [15]. Recently Kim et al. reported the glycolysis of PET waste into BHET using oyster shell-derived catalysts with a BHET yield of 68.6% at 195 °C [16]. Likewise, various other catalysts have been reported in the literature, including zeolites [17], deep eutectic solvents [18], ionic liquid-based glycolysis catalysts [19] (derived from Lewis acidic metal halides, acetates, carbonates, hydroxides, phosphates, and sulfates), metal oxides (Al, Fe, Co, Cu, and Zinc-based oxides) [20,21,22], These catalysts are reported to have moderate to excellent reactivity toward PET depolymerization. Nonetheless, the ease of availability and the expensive or toxic nature of these reported catalysts can be a hurdle for large-scale applications. Therefore, the pursuit of finding a more economical depolymerization catalytic system is still in progress. The most active glycolysis catalyst is zinc acetate, but as heavy metal, it has damaging effects on the environment because heavy metals are non-biodegradable which inspired Shukla and Kulkarni to implement mild alkali metal catalysts (sodium carbonate and sodium bicarbonate) for the depolymerization of PET waste. They found that the product yields (up to 62%) were almost comparable to those of conventional lead and zinc acetate catalysts [23]. The application of sodium carbonate for PET glycolysis has also been reported by López-Fonseca et al. achieving BHET yield close to 70% at 196 °C with an EG: PET molar ratio of 7.6 [24]. Sodium methoxide (MeONa) has also been studied for POSTC-PET waste hydrolysis to produce TPA in methanol and dimethyl sulfoxide (DMSO) as solvent under microwave irradiation [25]. There is a fundamental difference between catalysis by the Lewis-acidic metal acetates, which are directly involved in the ester cleavage [19], and catalysis by Brønsted-basic metal alkoxides (MeONa in this study) that prepare the ethylene glycol for the direct attack [26]. Numerous parameters simultaneously influence the glycolysis of PET, which can be efficiently and systematically examined by response surface methodology. Researchers have implemented various models for the design of experiments in PET glycolysis using zinc acetate [27,28,29], cobalt acetate [30] or manganese acetate [31], and sodium bicarbonate [32].

Herein, we describe our study on the glycolysis of POSTC-PET waste with sodium methoxide as a low-cost depolymerization catalyst. The influence of reaction temperature on the PET conversion was studied. To determine the optimum operating conditions, a statistical approach of response surface methodology was followed to simultaneously study the four operating parameters. Kinetics and reaction equilibria were also investigated to provide valuable data for the process development of PET glycolysis. For this purpose, glycolysis experiments were carried out in a batch reactor at various temperatures, and a simple reversible mathematical model consistent with experimental data was developed. The apparent activation energy was determined from the Arrhenius plot and the ceiling temperature was calculated via Gibbs’ free energy.

### Research Significance

To the best of our knowledge, sodium methoxide has not been reported so far as a catalyst for PET glycolytic depolymerization. The outstanding advantage of sodium methoxide is that it is more economical and environmentally benign compared to the reported catalysts. This certainly makes it a very interesting candidate for a large-scale PET depolymerization process. The main product obtained in this process is mainly the BHET monomer that can easily be integrated into existing standard production lines of PET, mixed with virgin raw material polymerized to PET. The BHET monomer obtained was analyzed and characterized for its confirmation.

## 2. Materials and Methods

### 2.1. Materials

Ethylene glycol (>99.5%) was purchased from Carl Roth, Germany. Sodium methoxide (MeONa) (95%) and cobalt acetate Co(OAc)_2_ (>99.9%) were purchased from Sigma Aldrich, Darmstadt, Germany. Zinc acetate Zn(OAc)_2_ (>99.9%) was purchased from Alfa Aesar, Kandel, Germany. HPLC grade, technical grade methanol, and isopropanol (rinsing agent) were purchased from VWR Darmstadt, Germany. Deuterated methanol for NMR analysis was purchased from Deutero, Kastellaun, Germany. Glass microfiber filters type GF/C and grade MN GF-1 were purchased from VWR, Darmstadt, Germany, and were used to filter PET residue and glycolysis product, respectively. Post-Consumer soft drink soda bottles were used as POSTC-PET waste sources. They were washed, cleaned, and crushed to use as a substrate as represented in Figure S1 (Supplementary Materials).

### 2.2. Depolymerization of POSTC-PET Waste

To use post-consumer PET bottles as a substrate, labels and caps were taken off, and the bottles were manually cut into smaller pieces and then washed. After washing, PET was shredded into small particles using a grinder. The crushed PET particles were placed into a sieving tower and sorted into various particle size fractions (0.25–1 mm). They were rinsed with isopropanol and stored after drying (60 °C overnight). Batch-wise glycolysis experiments were carried out in a glass reactor with a known quantity of PET of the desired particle size. EG and catalyst were added in specific ratios. The glass reactor containing PET, EG, and catalyst was then put onto a heating block with a temperature controller, for the desired time under constant magnetic stirring (500 rpm). At the end of the reaction time, the heating was stopped, and 10 mL of hot (90 °C) water was added to the mixture and filtered afterward. The reaction mixture was filtered using microfiber filter type GF/C under vacuum. In this way the reaction mixture was separated into two different fractions, (i) The solid residual fraction made up primarily of unconverted PET, named (A), and (ii) the liquid filtrate fraction consisting mainly of BHET, EG, and water, named (B). The residue fraction (A) was dried overnight in an oven at a temperature of 60 °C to determine the conversion of the initial PET substrate with the help of Equation (1):

(1) $$PET\;Conversion,\;X\;\left(\%\right)=\frac{m_{PET,0}-m_{PET,t}}{m_{PET,0}}\times 100$$

where $m_{PET,0}$ is the initial mass of PET in gram, $m_{PET,t}$ is the mass of incompletely depolymerized PET in gram after a certain reaction time t. To crystallize the product, the filtrate fraction (B) was stored overnight in the refrigerator at 4 °C. White crystalline BHET was filtered using glass microfiber filter grade MN GF-1 under vacuum. The solid crystals, named as a fraction (C) were then dried overnight in an oven at a temperature of 60 °C to analyze the product BHET.

As a precaution, the remaining filtrate was stored again in the refrigerator named as a fraction (D). The stored BHET was further analyzed to confirm the product. BHET yield was calculated using the following formula (Equation (2)):

(2) BHET Yield, Y (%)=mBHETMBHETmPET,0MPRU×100

where $m_{PET,0}$ is the initial weight of PET in gram, $m_{BHET}$ is the weight of BHET crystals collected in gram, $M_{BHET}$ is the molecular weight of BHET (254 g/mol) and $M_{PRU}$ is the molecular weight of the PET-Repeating-Unit (PRU) (192 g/mol). The experimental setup of PET glycolysis is shown in Figure S2 (Supplementary Materials).

Turn over number (TON) and Turn over frequency (TOF) of the catalysts were calculated using Equations (3) and (4) respectively. It should be noted that *TOF* is calculated for 20% conversion and is therefore described as *TOF*_20._

(3) $$TON=\frac{n\left(PETconverted\right)}{n\left(cat.\right)}$$

(4) $$TOF_{20}=\frac{TON}{\;time}$$

where *n(PET converted)* is the number of moles of *PET* substrate that are converted after the reaction, *n(cat.)* is the number of moles of catalyst used in the respective reaction, and time is in hrs. The reported standard procedure used to compare depolymerization results with literature results, i.e., the addition of water for BHET precipitation, will of course deactivate the catalyst (MeONa) by hydrolysis. Consequently, a new approach to extracting pure BHET product and efficiently reusing the catalyst is currently being developed in our laboratory.

### 2.3. Product Characterization

BHET product was analyzed by GC-MS, NMR, melting point, and DSC. DSC analysis was also used to analyze PET residues. The supporting information gives the corresponding details on these data.

## 3. Results and Discussion

### 3.1. Impact of Catalyst and Selection of Reaction Temperature

**Figure 1:**
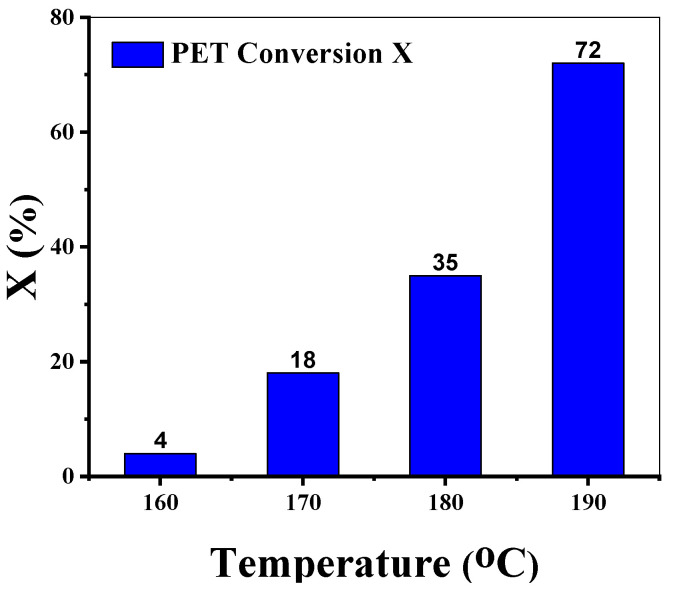
Temperature screening using MeONa as a glycolysis catalyst.

The impact of sodium methoxide (as a glycolysis catalyst) on PET conversion was studied. First, an uncatalyzed experiment was performed as a reference, where only 3% conversion was possible over 6 h at 190 °C. Almost all the substrate remained in the residue (see Supplementary Materials for complete reaction conditions). The result showed that the application of a catalyst is highly advisable. Afterward, the temperature was varied between 160 and 190 °C. The results are presented in Figure 1. The presumed strong temperature dependency was indeed found for sodium methoxide. A difference in the conversion of about 68% over 30 °C is remarkable. This significant influence of temperature for an increase in terephthalic acid (TPA) yield was also observed by Mohsin et al. [25] while using MeONa as a catalyst under microwave irradiation. Since the highest conversion was reached at 190 °C but the used experimental set-up is limited by the boiling point of EG (197–198 °C) [33,34], 190 °C was used for DoE optimization of the remaining glycolysis parameters. However, to compare the MeONa performance with the commonly used catalyst (Zn and Co acetate) at literature-reported conditions, further experiments were also performed just below the boiling point of EG. Those results are given in Section 3.4 (Performance Comparison for Sodium Methoxide).

Reaction conditions: T = 160–190 °C, PET: Cat = 100 (mol/mol), EG: PET = 5 (mol/mol), time = 4 h, PS = 1 mm

### 3.2. Analysis of Glycolysed Products

To confirm that the fraction (C) is the BHET monomer, GC-MS, ^1^H NMR, ^13^C NMR, melting point, and DSC analysis were performed. As shown in Figure S3 (Supplementary Materials), it is clear from the mass spectrum of fraction (C) that the most significant characteristic peaks are intense (at *m*/*z* 211.1, 193.1, 149, 121.1, and 104) and are attributed to the BHET monomer as reported in the literature [14,35]. ^13^C NMR of faction (C) is shown in Figure S4 in which the signal at 167.18 ppm indicates carbon of the carbonyl group (–COO–), and signals at 135.44 and 130.68 ppm indicate aromatic carbon associated with carbonyl groups and hydrogen respectively. The signal at 67.96 ppm indicates the presence of carbon in the methylene group along the carbonyl group and the signal at 61.06 ppm represented the carbon in the methylene group neighboring to the hydroxyl group (–OH). ^1^H NMR of fraction (C) is shown in Figure S5 in which the signal at 8.17 ppm indicates aromatic hydrogen, and the signal at 4.85 ppm indicates hydrogen of the hydroxyl group (–OH). The signal at 4.41 ppm indicates the presence of hydrogen in the methylene group along the carbonyl group and the signal at 3.88 ppm represented the hydrogen in the methylene group neighboring to the hydroxyl group (–OH). The NMR spectrum confirms the BHET as the main product of PET glycolysis and is consistent with the reported literature [16,36,37,38].

The melting point of commercial and produced BHET as well as of PET residue was measured using a melting point analyzer. The operating conditions and corresponding results are given in Table S1. The commercial BHET starts to melt at around 103.1 °C and melts completely up to 107.4 °C. On the other hand, produced BHET starts to melt slightly later around 107.8 °C but melts completely around 111.7 °C. The melting point of unconverted PET residue was also measured and it was found that residue starts melting around 162 °C but melts completely around 168 °C corresponding to the dimer melting temperature range [38]. The melting point of BHET is in accordance as reported in various studies 109–110 [39], 111 [40] and 113.34 °C [41] via DSC-analysis. The melting point analysis is further supported by DSC analysis (Figure S5) which shows that PET residue comprises only monomer and dimer peaks. The small melting peak at 107 °C indicates the presence of BHET monomer and is likely the result of the monomer precipitating with the dimer instead of being dissolved by the water during filtration. Therefore, it is apparent that glycolysis comprises a monomer-dimer equilibrium, and no PET substrate is present in the residue [42,43]. To validate this further, a series of control experiments were performed, varying the reaction time to understand the degradation mechanism. In Figure S6, three main DSC peaks were observed around 110, 150–160, and 250 °C, which were attributed to trace amounts of BHET (not completely extracted), dimers of different compositions, and unconverted PET respectively [44]. It can also be observed in Figure S6, that the peak related to PET remarkably decreased with degradation time and is attributed to an increase in the depolymerization rate and eventually the formation of BHET monomer.

### 3.3. Design of Experiments (DoE)

To study the combined effect of all parameters, the experimental plan was carried out through a response surface methodology (RSM) approach, based on Box-Behnken design by the software Design Expert. Four independent variables (PET: MeONa (molar ratio), EG: PET (molar ratio), reaction time, and particle size) were investigated with the help of 29 experiments. PET conversion X (%) was the observed response. The coded and uncoded values of independent factors along with their ranges are given in Table 1.

The experimental Box-Behnken design (BBD) matrix with the observed responses from 29 experimental runs is presented in Table S2 (Supplementary Materials) and was further used to find the regression model after inserting the observed responses. Analysis of variance (ANOVA) was performed to find the significant parameters with the help of a probability p-value test. The coefficient of determination (R^2^), adjusted R^2^, adequate precision, and test for Lack of Fit were determined to check the correctness of the established model. A second-order model (Equation (5)) was selected to describe the observed response (PET conversion X):

(5) $$PET\;Conversion\;\left(X\right)=b_{o}+\overset{n}{\underset{i=1}{\sum }}b_{i}x_{i}+\overset{n}{\underset{i=1}{\sum }}b_{ii}x_{i}^{2}+\overset{n-1}{\underset{i=1}{\sum }}\overset{n}{\underset{j=i+1}{\sum }}b_{ij}x_{i}x_{j}$$

In Equation (5), *X* is the predicted response for *PET Conversion*, *b_o_* is the intercept term, and linear and second-order polynomial coefficients are represented by *b_i_* and *b_ii_* respectively. Interaction terms are given by *b_ij_* whereas *x_i_* and *x_j_* are the coded independent variables. n is the number of independent variables which in this case is four as given in Table 2. The relationship between the coded factors (*C_i_*) and the actual values (*V_i_*) is given by the following generalized expression (Equation (6)):

(6) $$C_{i}=\frac{V_{i}-M_{i}}{H_{i}-M_{i}}$$

#### 3.3.1. Development of Regression Model from Design Expert

Experimental data for PET conversion was fitted with a quadratic model. The suitability of the models was tested by the “coefficient of determination” (R^2^), which is a measure of the fitting degree. A good fitting model was obtained for PET conversion, where the R^2^ value is greater than 95%. Table 2 shows the important fit statistics for the fitted model.

The value of R^2^ shows the correlation between values predicted by the model and values observed experimentally. The R^2^ value of 0.9552 means that more than 95% of the experimental points will lie within the regression line. “Adeq Precision” measures the signal-to-noise ratio. A ratio greater than 4 is desirable [45]. The adequate signal, in this case, is >19 (Table 2), indicating an adequate signal. Therefore, the fitted model can be used to navigate the design space. The fit summary of the tested models is also provided in Table S3 (Supplementary Materials), which gives the comparison of all the available models, and the criteria for the selection of the model is also explained in Supplementary Materials. The ANOVA results are also given in Table S4. If the *p*-value is lower than 0.0500 [46], it indicates that model and model terms are significant and the values greater than 0.1000 indicate that the terms are insignificant (Table S4). The Lack of Fit (*p*-value) implies that the Lack of Fit is not significant as the values are greater than 0.0500. Non-significant Lack of Fit is good for model fitting. The significant terms are given in Table 2. The coefficients of the second-order polynomial (quadratic) equation are also given in Table S4 (Supplementary Materials). Based on regression coefficients given in Table S4, the mathematical quadratic model for PET conversion in terms of significant terms is given in Equation (7). However, the complete quadratic model, including insignificant terms, is also given in Supplementary Equation (S1) (Supplementary Materials).

(7) $$PET\;Conversion\;\left(X\right)=68.53-6.98\times C_{1}+11.78\times C_{2}+10.39\times C_{3}-5.17\times C_{1}\times C_{2}+6.84\times C_{1}\times C_{3}+4.33\times C_{1}\times C_{4}-7.84\times C_{3}^{2}$$

**Figure 2:**
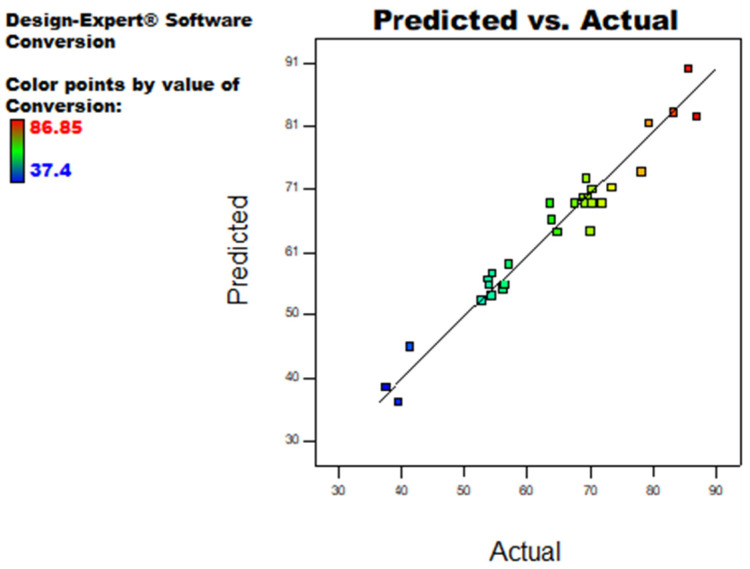
Performance and behavior of Equation (7) are plotted against predicted values and actual (experimental) values.

A comparison of predicted versus actual values of measured response (PET Conversion) is presented in Figure 2. It can be seen that in most experimental runs, values predicted by the model are very close to the actual experimental values, which is in strong agreement with a higher value of R^2^ (>0.95). Therefore, the model can be used to navigate the design space as it is appropriate to explain the relationship between the independent variables and the observed response. The maximum PET conversion achieved within the design space by sodium methoxide to depolymerize POSTC-PET waste was recorded as 86%.

#### 3.3.2. Interactive Effects

**Figure 3:**
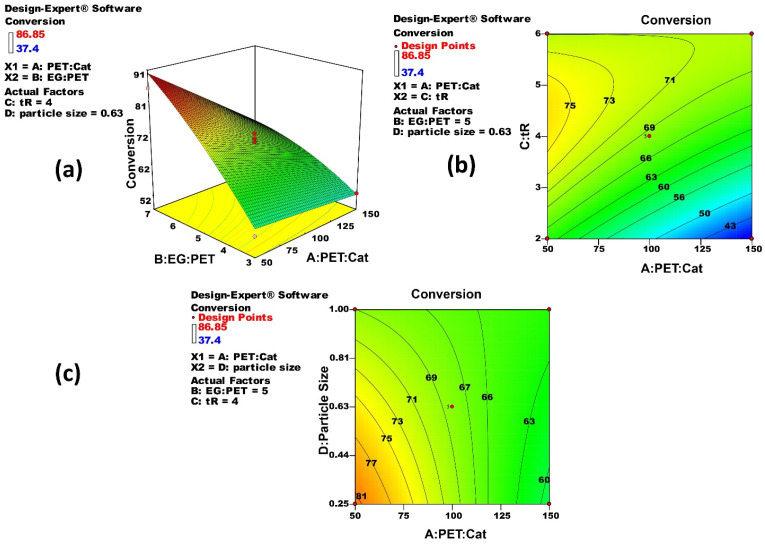
Response surface and contour plots of significant terms from DoE illustrate the performance and behavior of Equation (7).

The interaction effect of significant terms (AB, AC, and AD) is shown in Figure 3 and the following interpretations can be made (i) An excess EG is required for good catalytic performance (Figure 3a) as EG acts as a solvent and its higher concentration will shift the equilibrium towards the product side, the monomer BHET (Scheme 1), (ii) The combination of a low catalyst concentration and a short reaction time always results in low conversion (Figure 3b). While it is rather safe to assume that a longer reaction time will always yield a higher conversion, however, the maximum concentration is not at high levels of reaction time (Figure 3b). It means the optimum lies below the high levels of reaction time, (iii) A combined effect of lower particle size and higher catalyst concentration promotes high conversion (Figure 3c). The smaller particles possess larger specific surfaces which accelerate the dissolution speed in EG resulting in higher conversions [24], (iv) These observations allow us to sort the effects of significant parameters on measured response in the order of B > C > A > C^2^ > A × C > A × B > A × D.

#### 3.3.3. Model Optimization

Based on the obtained experimental results so far (Table S2), the next step is the optimization of all the independent parameters to achieve maximum PET conversion. The list of a tentative optimum set of reaction parameters is provided in Table S5. Since the desirability function of entries, 1 and 2 are similar (Figures S8 and S9) and entry 2 allows for a shorter reaction time, this parameter set was selected as the optimum. The regression model predicts 87% PET conversion at 190 °C, a reaction time of 2.1 h, PET: MeONa of 53.86, EG: PET = 6.98, and a particle size of 0.25 mm. These optimized conditions are milder as compared to those already reported in the literature [47]. The optimization experiment was performed 3 times and the experimental average PET conversion (82%) was found to be with a slight deviation of approximately 5% from the predicted response. The isolated BHET yield at the optimum conditions was found to be 65%. Hence, the regression model is adequate in predicting the response in the investigated design space.

### 3.4. Performance Comparison for Sodium Methoxide

**Figure 4:**
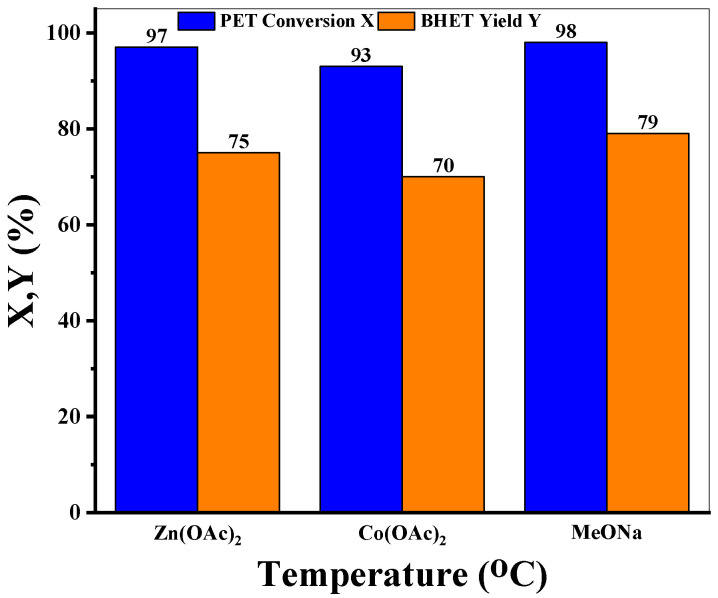
Performance comparison of MeONa with different catalysts.

The glycolysis performance of sodium methoxide was compared to widely studied zinc acetate and cobalt acetate at operating conditions as reported in the literature [47]. From Figure 4, it is obvious that MeONa has comparable depolymerization efficiency (PET conversion of 98%, BHET isolated yield of 79%) as compared to zinc acetate (PET conversion 97%, BHET isolated yield 75%) and cobalt acetate (PET conversion 93%, BHET isolated yield 70%). The TON obtained for sodium methoxide (39) is very similar to the TON obtained with zinc acetate (40) and cobalt acetate (40) under the given conditions (Table S6). However, the TOF_20_ values show a wider spread, i.e., 314 for zinc acetate, 58 for sodium methoxide, and 54 for cobalt acetate. On the other hand, sodium methoxide is inexpensive (cost comparison in Table S6) [48,49,50] and more environmentally benign as compared to zinc acetate and cobalt acetate, which certainly makes it a more feasible option for a large-scale PET depolymerization process.

Reaction conditions: T = 197 °C, time = 1.5 h, PET: Cat = 26 (mol/mol), EG: PET = 13.74 (mol/mol), PS = 0.125 mm, rpm = 500.

### 3.5. Thermodynamic and Kinetic Evaluation

#### 3.5.1. Van’t Hoff Plot

**Figure 5:**
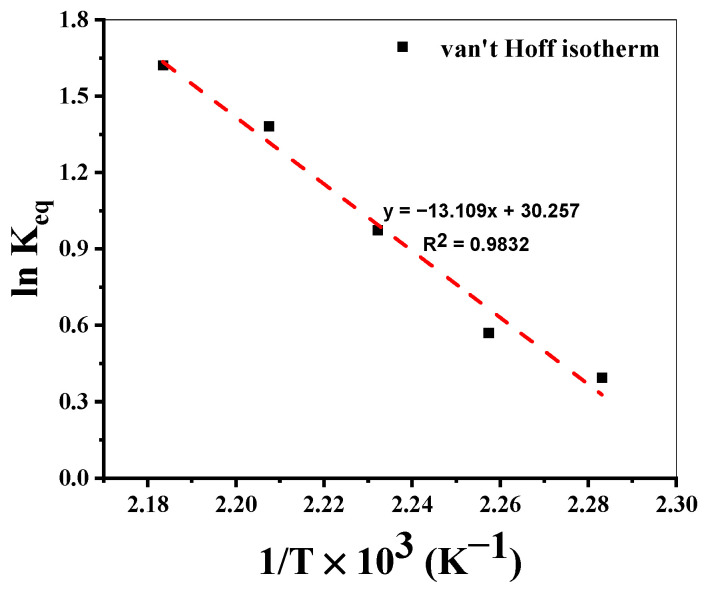
van’t Hoff plot for evaluation of thermodynamic parameters.

Glycolysis experiments were performed to measure the thermodynamic parameters to ensure equilibrium was reached. Figure 5 shows the van’t Hoff plot based on Equation (8). Equilibrium constants ($K_{eq})$ at the respective temperatures were calculated using Equation (9).

(8) $$\ln K_{eq}=-\left(\frac{\Delta H}{R}\right)\frac{1}{T}+\frac{\Delta S}{R}$$

(9) $$K_{eq}=\frac{x_{BHET,eq}}{x_{PET,\;eq}.x_{EG,eq}}$$

(10) $$T_{C}\;\left[K\right]={\frac{\Delta H_{R}}{\Delta S_{R}}|}_{\;\Delta G=0}$$

The van’t Hoff plot shows a typical endothermic behavior. The reaction enthalpy calculated from the slope is Δ*H* = 109 kJ/mol. An entropy calculated from the intercept is Δ*S* = 252 J/mol^.^K. The ceiling temperature (*T_C_*) calculated from Equation (10) is found to be 160 °C, for equilibrium reaction Δ*G_R_* = 0. To validate this idea, at 150 °C (*T* < *T_C_*), PET conversion of only 9% was achieved even after 6 h, indicating the necessity of *T* > *Tc*.

Reaction Conditions: T = 165–185 °C, PET:Cat = 50 (mol/mol), EG:PET = 7 (mol/mol), PS = 0.25 mm.

#### 3.5.2. Reaction Kinetics

The kinetics of PET glycolysis is described by a simple reversible model, assuming a homogeneous reaction and considering the monomer-dimer equilibrium in Scheme 1, including the backward reaction. The following equation defines the catalytic depolymerization rate,

(11) $$-\frac{d\left[PRU\right]}{dt}=k_{G,1}\left[cat\right]\left[EG\right]\left[PRU\right]-k_{G,2}\left[cat\right]\left[BHET\right]$$

The terms are named as before, the only differences are the split of $k_{G}$ into forward (1) and backward (2) reactions, and [BHET] being the concentration of the formed bis(2-hydroxyethyl) terephthalate. The $k_{G,1}$ and and $k_{G,2}$ will be again lumped with the constant catalyst and EG concentration, and named $k_{G,1}^{’}$ and $k_{G,2}^{’}$. After introducing *X_eq_* (equilibrium conversion) followed by the separation of variables and integration, the linear form of a pseudo-first-order kinetic model for a reversible reaction is obtained as follows.

(12) $$-\frac{d\left[PRU\right]}{dt}=k_{G,1}\left[cat\right]\left[EG\right]\left[PRU\right]-k_{G,2}\left[cat\right]\left[BHET\right]$$

#### 3.5.3. Apparent Activation Energy

The well-known Arrhenius equation is used to find the apparent activation energy.

(13) $$k=k_{o}\exp\left(-\frac{E_{A}}{RT}\right)$$

**Figure 6:**
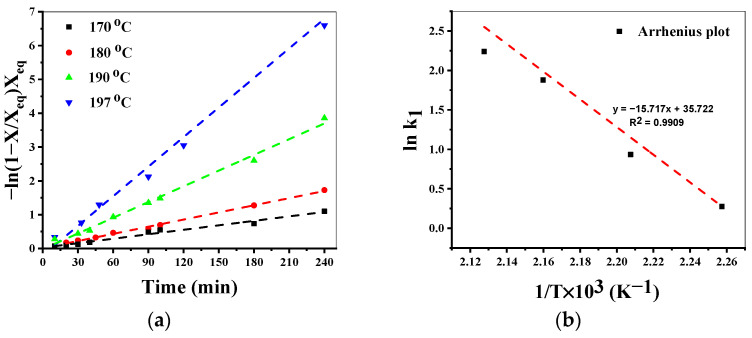
Kinetic model fitting of PET glycolysis in homogenous system (**a**) effect of temperature on reaction rate (**b**) Arrhenius plot for the homogenous system.

Kinetic data for the pseudo-first-order kinetic model (Equation (12)) is presented in Figure 6a. The homogenous reversible model showed a good fit in the temperature range of 170–197 °C. The linear correlation factor (R^2^) is above 0.98 for all the studied temperatures. Arrhenius plot for the homogenous reversible model also shows excellent graphical linearity (Figure 6b) with R^2^ above 0.99. The calculated energy of activation *E*_*A*_, is 130 kJ/mol for the depolymerization reaction and is far lower than the *E*_*A*_ reported for sodium carbonate (185 kJ/mol) [51] by applying a similar homogeneous reversible catalytic model.

Reaction conditions: Catalyst: MeONa, T = 170–197 °C, PET:Cat = 50 (mol/mol), EG:PET = 7 (mol/mol), PS = 0.25 mm.

The scope of this depolymerization study was to evaluate the depolymerization performance of environmentally benign sodium methoxide catalyst in the presence of ethylene glycol. Heavy metal ions are dangerous polluting agents as they are non-biodegradable and the toxicity caused by them is slow and lifelong [23]. Based on the results as discussed, MeONa has the potential to use as an environment-friendly alternative to heavy metal catalysts, having good depolymerization performance for post-consumer PET waste.

## 4. Conclusions

PET can be depolymerized into BHET in the presence of sodium methoxide utilizing ethylene glycol as a reagent and solvent. PET conversion is dependent on various factors including reaction temperature and time, the substrate-to-catalyst ratio, solvent-to-substrate ratio, and the size of PET particles. Therefore, response surface methodology, based on the Box-Behnken model, was used to study these four independent factors. Analysis of variance was performed and based on fit statistics, the developed regression model is adequate to predict the PET conversion. The regression models and the interactive study of parameters reveal that the order of significance of the parameters is as follows: EG > reaction time > catalyst loading > particle size. It was also found that an excess of ethylene glycol greatly enhances the depolymerization performance. The optimum conversion (87%) was found to be at 190 °C, EG: PET= 6.98 mol/mol, 2.11 h, PET: MeONa = 53.86 (mol/mol), and particle size 0.25 mm. The ceiling temperature (*T_C_* = 160 °C) was calculated from Gibbs’ free energy and the reaction enthalpy of the glycolysis reaction was found to be 109 kJ/mol. Kinetic investigations revealed that the depolymerization reaction follows a pseudo-first-order reversible reaction and the activation energy for MeONa was estimated as *E_A_* = 130 kJ/mol. DSC analysis confirmed that degradation of the substrate occurs from the terminal groups and leads to the formation of dimer and finally monomer. Under similar conditions, the catalytic depolymerization efficiency of MeONa (PET conversion 98%, BHET yield 79%). was also compared to that of zinc acetate (PET conversion 97%, BHET yield 75%) and cobalt acetate (PET conversion 93%, BHET yield 70%). All compared catalysts gave virtually complete conversion. The huge advantage of sodium methoxide is that it is way cheaper and more environmentally benign than heavy metal catalysts. This makes it a very interesting catalyst candidate for further PET depolymerization process development.

## 5. Recommendation and Outlook

The addition of water for BHET precipitation will deactivate the catalyst (MeONa) by hydrolysis. Therefore, at present, we are working on a new approach for BHET recovery without the addition of water. This will be key to further development, including the reuse of catalyst and excess EG.

## Figures and Tables

**Table 1:** Coded and uncoded values of independent variables.

Independent Variable	Symbol		Coded Levels		
	Actual (Vi)	Coded (Ci)	−1 (L)	0 (M)	+1 (H)
PET:MeONa (mol/mol)	A	C1	50	100	150
EG:PET (mol/mol)	B	C2	3	5	7
Reaction time, (h)	C	C3	2	4	6
Particle size, (mm)	D	C4	0.25	0.625	1

**Table 2:** Statistics parameters for the regression model (Equation (7)).

Response	PET Conversion (X)
R^2^	0.9552
Adjusted R^2^	0.9103
Predicted R^2^	0.7770
Adequate Precision (>4)	19.288
Lack of Fit (*p*-value)	1.73
Significant terms	A, B, C, AB, AC, AD, C^2^

## Data Availability

Not applicable.

## Supplementary Materials

The following supporting information is available at: https://www.mdpi.com/article/10.3390/polym15030687/s1. Figure S1. Substrate preparation for glycolysis. Figure S2. Glycolysis procedure in the laboratory. Figure S3. Mass spectrum of produced BHET. Figure S4. ^13^C NMR spectrum of produced BHET. Figure S5. ^1^H NMR spectrum of produced BHET. Figure S6. DSC scan of reaction residue. Figure S7. DSC scan of reaction residue with time variations. Figure S8. Desirability function of optimum solution 1. Figure S9. Desirability function of optimum solution 2. Table S1. Melting temperatures of produced and commercial BHET. Table S2. Experimental layout and output responses based on Box-Behnken design. Table S3. Fit Summary of the tested models for PET Conversion X. Table S4. ANOVA summary and coefficients of the second-order polynomial (quadratic) equation. Table S5. List of the possible optimum set of parameters after DoE optimization. Table S6. Cost comparison of sodium methoxide.

## References

[1] Suhaimi N.A.S., Muhamad F., Abd Razak N.A., Zeimaran E. (2022). Recycling of polyethylene terephthalate wastes: A review of technologies, routes, and applications. Polym. Eng. Sci..

[2] Volmajer Valh J., Stopar D., Selaya Berodia I., Erjavec A., Šauperl O., Fras Zemljič L. (2022). Economical Chemical Recycling of Complex PET Waste in the Form of Active Packaging Material. Polymers.

[3] MacDonald W.A. (2002). New advances in poly(ethylene terephthalate) polymerization and degradation. Polym. Int..

[4] Statista PET Global Production 2020|Statista. https://www.statista.com/statistics/650191/global-polyethylene-terephthalate-production-outlook/.

[5] Benyathiar P., Kumar P., Carpenter G., Brace J., Mishra D.K. (2022). Polyethylene Terephthalate (PET) Bottle-to-Bottle Recycling for the Beverage Industry: A Review. Polymers.

[6] Statista PET Global Bottle Production 2021|Statista. https://www.statista.com/statistics/723191/production-of-polyethylene-terephthalate-bottles-worldwide/.

[7] Nabid M.R., Bide Y., Jafari M. (2019). Boron nitride nanosheets decorated with Fe3O4 nanoparticles as a magnetic bifunctional catalyst for post-consumer PET wastes recycling. Polym. Degrad. Stab..

[8] Tuna Ö., Bal A., Güçlü G. (2013). Investigation of the effect of hydrolysis products of postconsumer polyethylene terephthalate bottles on the properties of alkyd resins. Polym. Eng. Sci..

[9] Al-Sabagh A.M., Yehia F.Z., Eshaq G., Rabie A.M., ElMetwally A.E. (2016). Greener routes for recycling of polyethylene terephthalate. Egypt. J. Pet..

[10] Mishra S., Zope V., Goje A. (2003). Kinetics and thermodynamics of hydrolytic depolymerization of poly(ethylene terephthalate) at high pressure and temperature. J. Appl. Polym. Sci..

[11] Grause G., Handa T., Kameda T., Mizoguchi T., Yoshioka T. (2011). Effect of temperature management on the hydrolytic degradation of PET in a calcium oxide filled tube reactor. Chem. Eng. J..

[12] Pang K., Kotek R., Tonelli A. (2006). Review of conventional and novel polymerization processes for polyesters. Prog. Polym. Sci..

[13] Baliga S., Wong W.T. (1989). Depolymerization of poly(ethylene terephthalate) recycled from post-consumer soft-drink bottles. J. Polym. Sci. A Polym. Chem..

[14] Ghaemy M., Mossaddegh K. (2005). Depolymerisation of poly(ethylene terephthalate) fibre wastes using ethylene glycol. Polym. Degrad. Stab..

[15] Fehér Z., Kiss J., Kisszékelyi P., Molnár J., Huszthy P., Kárpáti L., Kupai J. (2022). Optimisation of PET glycolysis by applying recyclable heterogeneous organocatalysts. Green Chem..

[16] Kim Y., Kim M., Hwang J., Im E., Moon G.D. (2022). Optimizing PET Glycolysis with an Oyster Shell-Derived Catalyst Using Response Surface Methodology. Polymers.

[17] Shukla S.R., Palekar V., Pingale N. (2008). Zeolite catalyzed glycolysis of poly(ethylene terephthalate) bottle waste. J. Appl. Polym. Sci..

[18] Sert E., Yılmaz E., Atalay F.S. (2019). Chemical Recycling of Polyethlylene Terephthalate by Glycolysis Using Deep Eutectic Solvents. J. Polym. Env..

[19] Al-Sabagh A.M., Yehia F.Z., Eissa A.M.F., Moustafa M.E., Eshaq G., Rabie A.M., ElMetwally A.E. (2014). Cu- and Zn-acetate-containing ionic liquids as catalysts for the glycolysis of poly(ethylene terephthalate). Polym. Degrad. Stab..

[20] Jeya G., Dhanalakshmi R., Anbarasu M., Vinitha V., Sivamurugan V. (2022). A short review on latest developments in catalytic depolymerization of Poly (ethylene terephathalate) wastes. J. Indian Chem. Soc..

[21] Ghosal K., Nayak C. (2022). Recent advances in chemical recycling of polyethylene terephthalate waste into value added products for sustainable coating solutions–hope vs. hype. Mater. Adv..

[22] Xin J., Zhang Q., Huang J., Huang R., Jaffery Q.Z., Yan D., Zhou Q., Xu J., Lu X. (2021). Progress in the catalytic glycolysis of polyethylene terephthalate. J. Environ. Manage..

[23] Shukla S.R., Kulkarni K.S. (2002). Depolymerization of poly(ethylene terephthalate) waste. J. Appl. Polym. Sci..

[24] López-Fonseca R., Duque-Ingunza I., de Rivas B., Arnaiz S., Gutiérrez-Ortiz J.I. (2010). Chemical recycling of post-consumer PET wastes by glycolysis in the presence of metal salts. Polym. Degrad. Stab..

[25] Mohsin M.A., Alnaqbi M.A., Busheer R.M., Haik Y. (2018). Sodium Methoxide Catalyzed Depolymerization of Waste Polyethylene Terephthalate Under Microwave Irradiation. Catal. Ind..

[26] Aziz N.A.M., Hamid H.A., Yunus R., Abbas Z., Omar R., Rashid U., Syam A.M. (2020). Kinetics and thermodynamics of synthesis of palm oil-based trimethylolpropane triester using microwave irradiation. J. Saudi Chem. Soc..

[27] Aguado A., Martínez L., Becerra L., Arieta-araunabeña M., Arnaiz S., Asueta A., Robertson I. (2014). Chemical depolymerisation of PET complex waste: Hydrolysis vs. glycolysis. J. Mater. Cycles Waste Manag..

[28] Stoski A., Viante M.F., Nunes C.S., Muniz E.C., Felsner M.L., Almeida C.A.P. (2016). Oligomer production through glycolysis of poly(ethylene terephthalate): Effects of temperature and water content on reaction extent. Polym. Int..

[29] Katoch S., Sharma V., Kundu P.P., Bera M.B. (2012). Optimization of PET Glycolysis Process by Response Surface Methodological Approach: A Two-Component Modelling Using Glycolysis Time and Temperature. ISRN Polym. Sci..

[30] Chen C.-H., Chen C.-Y., Lo Y.-W., Mao C.-F., Liao W.-T. (2001). Studies of glycolysis of poly(ethylene terephthalate) recycled from postconsumer soft-drink bottles. II. Factorial experimental design. J. Appl. Polym. Sci..

[31] Chen C.-H. (2003). Study of glycolysis of poly(ethylene terephthalate) recycled from postconsumer soft-drink bottles. III. Further investigation. J. Appl. Polym. Sci..

[32] Van-Pham D.-T., Le Q.-H., Lam T.-N., Nguyen C.-N., Sakai W. (2020). Four-factor optimization for PET glycolysis with consideration of the effect of sodium bicarbonate catalyst using response surface methodology. Polym. Degrad. Stab..

[33] ChemicalBook Ethylene Glycol|107-21-1. https://www.chemicalbook.com/ChemicalProductProperty_EN_CB7852707.htm.

[34] Yue H., Zhao Y., Ma X., Gong J. (2012). Ethylene glycol: Properties, synthesis, and applications. Chem. Soc. Rev..

[35] Yunita I., Putisompon S., Chumkaeo P., Poonsawat T., Somsook E. (2019). Effective catalysts derived from waste ostrich eggshells for glycolysis of post-consumer PET bottles. Chem. Pap..

[36] Froidevaux V., Negrell C., Caillol S., Pascault J.-P., Boutevin B. (2016). Biobased Amines: From Synthesis to Polymers; Present and Future. Chem. Rev..

[37] Lima G.R., Monteiro W.F., Ligabue R., Santana R.M.C. (2017). Titanate Nanotubes as New Nanostrutured Catalyst for Depolymerization of PET by Glycolysis Reaction. Mat. Res..

[38] Xi G., Lu M., Sun C. (2005). Study on depolymerization of waste polyethylene terephthalate into monomer of bis(2-hydroxyethyl terephthalate). Polym. Degrad. Stab..

[39] Guclu G., Kasgoz A., Ozbudak S., Ozgumus S., Orbay M. (1998). Glycolysis of poly(ethylene terephthalate) wastes in xylene. J. Appl. Polym. Sci..

[40] Silva C.V.G., Silva Filho E.A.d., Uliana F., Jesus L.F.R.d., Melo C.V.P.d., Barthus R.C., Rodrigues J.G.A., Vanini G. (2018). PET glycolysis optimization using ionic liquid [Bmin]ZnCl3 as catalyst and kinetic evaluation. Polímeros.

[41] Veregue F.R., Pereira da Silva C.T., Moisés M.P., Meneguin J.G., Guilherme M.R., Arroyo P.A., Favaro S.L., Radovanovic E., Girotto E.M., Rinaldi A.W. (2018). Ultrasmall Cobalt Nanoparticles as a Catalyst for PET Glycolysis: A Green Protocol for Pure Hydroxyethyl Terephthalate Precipitation without Water. ACS Sust. Chem. Eng..

[42] Al-Sabagh A.M., Yehia F.Z., Eissa A.-M.M.F., Moustafa M.E., Eshaq G., Rabie A.-R.M., ElMetwally A.E. (2014). Glycolysis of Poly(ethylene terephthalate) Catalyzed by the Lewis Base Ionic Liquid [Bmim][OAc]. Ind. Eng. Chem. Res..

[43] Wang H., Liu Y., Li Z., Zhang X., Zhang S., Zhang Y. (2009). Glycolysis of poly(ethylene terephthalate) catalyzed by ionic liquids. Eur. Polym. J..

[44] Kratofil Krehula L., Hrnjak-Murgić Z., Jelenčić J., Andričić B. (2009). Evaluation of Poly(ethylene-terephthalate) Products of Chemical Recycling by Differential Scanning Calorimetry. J. Polym. Env..

[45] Chamoli S. (2015). ANN and RSM approach for modeling and optimization of designing parameters for a V down perforated baffle roughened rectangular channel. Alex. Eng. J..

[46] Rana A.G., Minceva M. (2021). Analysis of Photocatalytic Degradation of Phenol with Exfoliated Graphitic Carbon Nitride and Light-Emitting Diodes Using Response Surface Methodology. Catalysts.

[47] Goje A.S., Mishra S. (2003). Chemical Kinetics, Simulation, and Thermodynamics of Glycolytic Depolymerization of Poly(ethylene terephthalate) Waste with Catalyst Optimization for Recycling of Value Added Monomeric Products: 29. Macromol. Mater. Eng..

[48] Sodium Methoxide | Sigma-Aldrich, 2023.000Z. https://www.sigmaaldrich.com/DE/en/search/sodium-methoxide?focus=products&page=1&perpage=30&sort=relevance&term=sodium%20methoxide&type=product.

[49] Cobalt Acetate | Sigma-Aldrich, 2023.000Z. https://www.sigmaaldrich.com/DE/en/search/cobalt-acetate?focus=products&page=1&perpage=30&sort=relevance&term=cobalt%20acetate&type=product.

[50] Zinc Acetate | Sigma-Aldrich, 2023.000Z. https://www.sigmaaldrich.com/DE/en/search/zinc-acetate?focus=products&page=1&perpage=30&sort=relevance&term=zinc%20acetate&type=product.

[51] López-Fonseca R., Duque-Ingunza I., de Rivas B., Flores-Giraldo L., Gutiérrez-Ortiz J.I. (2011). Kinetics of catalytic glycolysis of PET wastes with sodium carbonate. Chem. Eng. J..

