# Supplementary material for: Optimization and Kinetic Evaluation for Glycolytic Depolymerization of Post-Consumer PET Waste with Sodium Methoxide
Source: Polymers (Basel). 2023 Jan 29;15(3):687. doi: 10.3390/polym15030687 (PMC9921498; doi:10.3390/polym15030687)
Supplement: Supplementary file 1 [file polymers-15-00687-s001.zip › polymers-2141548-supplementary.pdf]

Supporting Information

Optimization and Kinetic Evaluation for Glycolytic Depolymerization of Post-Consumer PET Waste with Sodium Methoxide

Saqib Javed [1,2], Jonas Fisse [1] and Dieter Vogt [1,*]

[1] Laboratory of Industrial Chemistry, Department of Biochemical and Chemical Engineering, TU Dortmund University, Emil-Figge-Straße 66, 44227 Dortmund, Germany
[2] Department of Chemical, Polymer, and Composite Materials Engineering, University of Engineering and Technology (UET), Lahore 39161, Pakistan
* Correspondence: dieter.vogt@tu-dortmund.de; Tel.: +49-231-755-2317

Contents

Substrate preparation for glycolysis 2
Glycolysis procedure 2
Uncatalyzed PET glycolysis experiment 3
Product Characterization 3
  GC-MS 3
  ^{13}C NMR 4
  ^{1}H NMR 5
  Melting Point Determination 6
  DSC 6
Design of Experiments (DoE) 8
DoE Model optimization 11
Performance comparison of sodium methoxide 13
List of Abbreviations 13

Substrate preparation for glycolysis

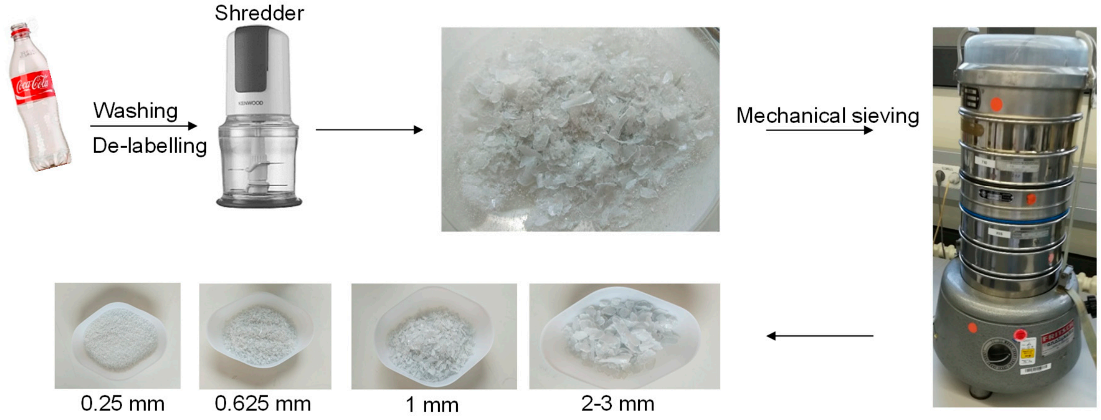

Figure S1: Substrate preparation for glycolysis

Glycolysis procedure

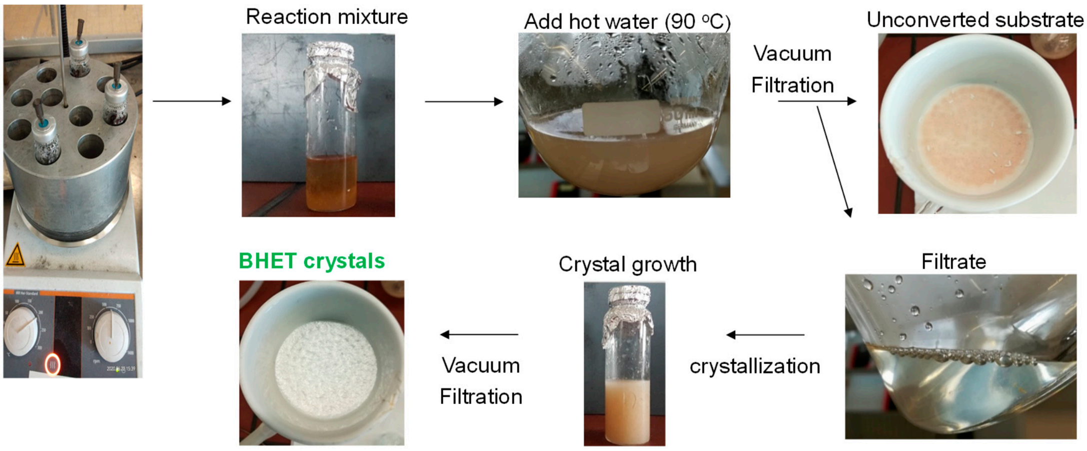

Figure S2: Glycolysis procedure in the laboratory

Uncatalyzed PET glycolysis experiment

Reaction conditions: T = 190 ºC, PET: Cat = -- (mol/mol), EG: PET = 7 (mol/mol), time =6 h, rpm= 500, PS = 1 mm

Results: PET conversion X (%) = 3% ± 5%

Note: Uncatalyzed experiments were performed 3 times and average conversion is reported.

Product Characterization

BHET product was characterized by GC-MS, NMR, DSC, and melting point analyzer. The mass spectrum was recorded after dilution with technical-grade methanol and NMR spectra were recorded after dilution with deuterated methanol. The melting point was measured using a solid sample of BHET without any pre-treatment.

GC-MS

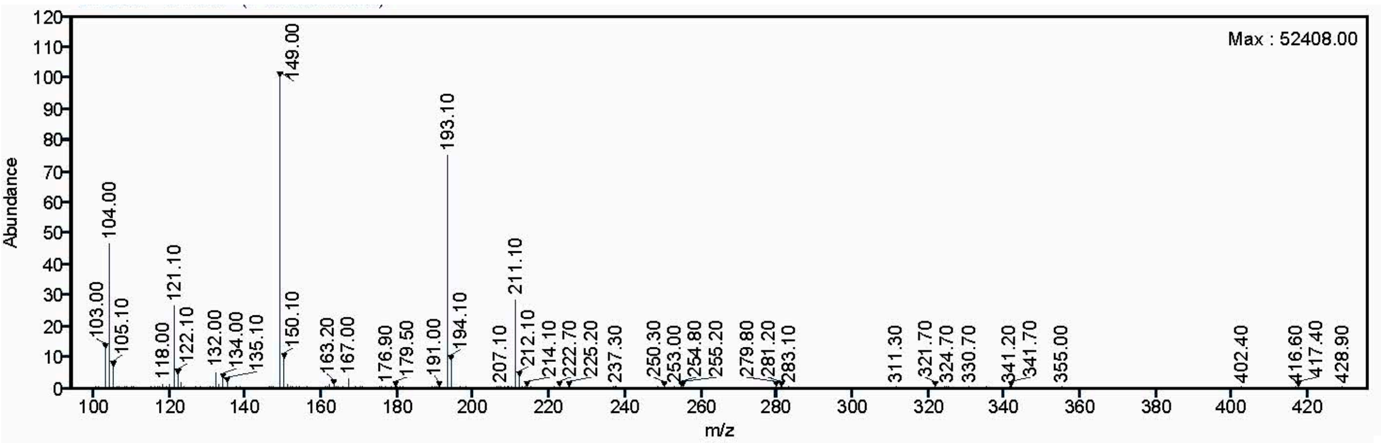

Figure S3: Mass spectrum of produced BHET

GC-MS: m/z (%) = 149(100), 193.1(75), 104(46), 211.1(28), 121.1(26), 103(12), 150(9), 194.1(9), 105.1(7), 132.1(5), 122.1(4), 212.1(3), 134(3), 167(3), 135.1(2.07), 123(1).

^{13}C NMR

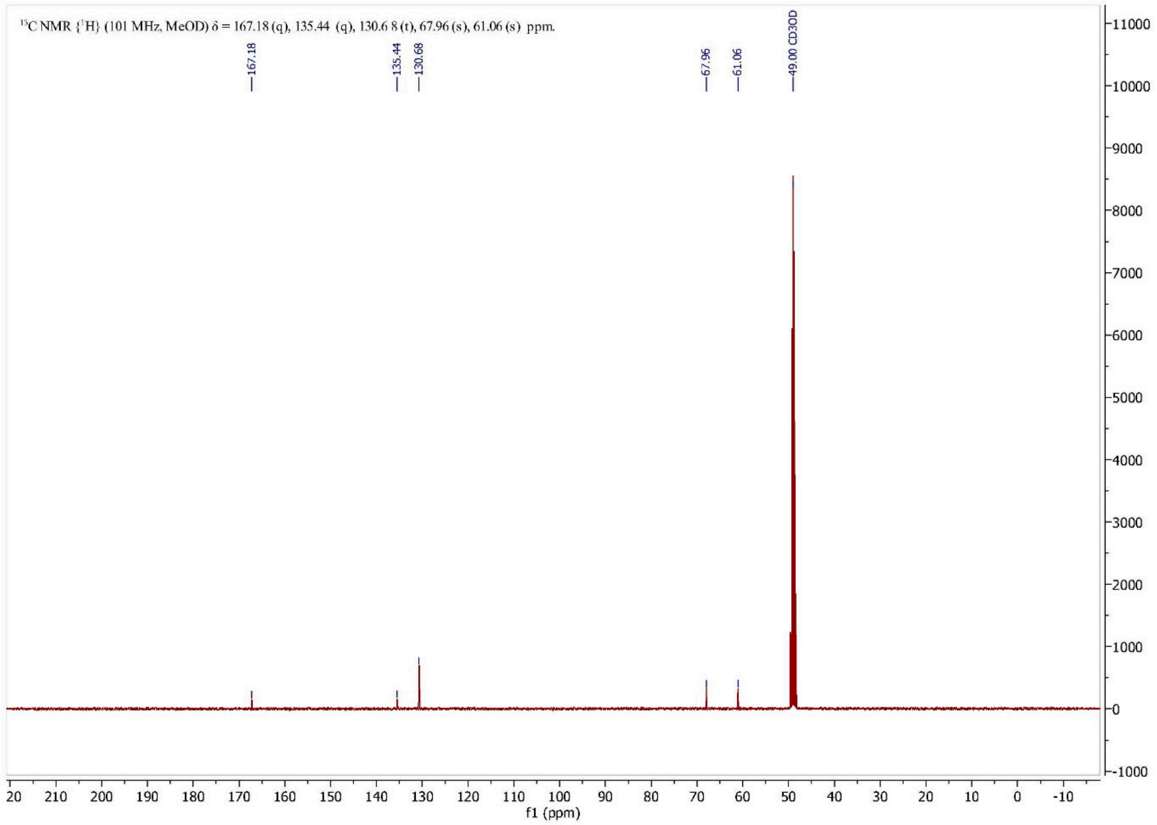

Figure S4: ^{13}C NMR spectrum of produced BHET

^{13}C NMR {1H} (101 MHz, Methanol-d4): δ = 167.18 (q), 135.44 (q), 130.6 8 (t), 67.96 (s), 61.06 (s) ppm.

^{1}H NMR

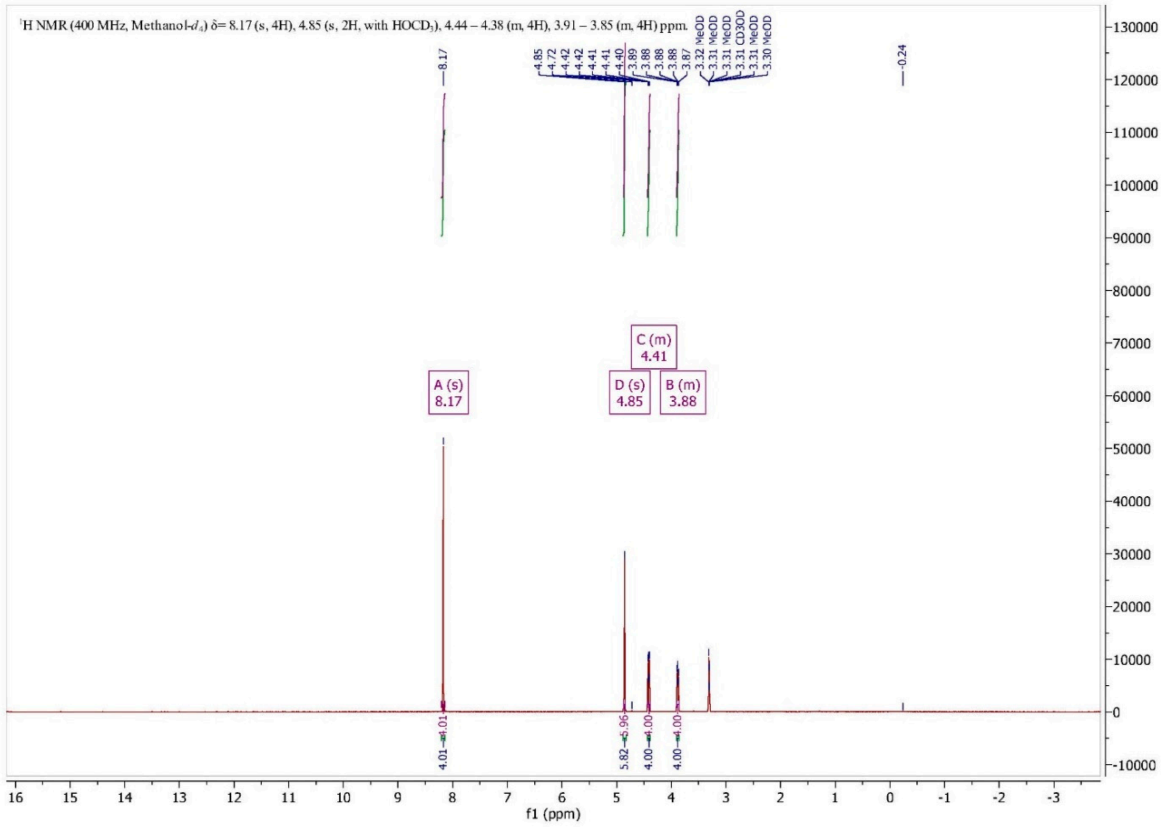

Figure S5: ^{1}H NMR spectrum of produced BHET

^{1}H NMR (400 MHz, Methanol-d4): δ = 8.17 (s, 4H), 4.85 (s, 2H, with HOCD3), 4.44 – 4.38 (m, 4H), 3.91 – 3.85 (m, 4H) ppm.

Melting Point Determination

The melting point of BHET and PET residue was measured in capillaries using a Melting Point analyzer. The different samples were analyzed according to their (expected) properties. Operating conditions and corresponding results are given in Table 1S.

Table S1: Melting temperatures of produced and commercial BHET

Sample Name	Operating conditions			Measurement /Sample No.	Melting starts (°C)	Melting ends (°C)
	T_{Start} [°C]	T_{End} [°C]	Heating ramp [°C/min]			
BHET Produced	50	150	3	1	107.8	111.7
				2	108.5	110.9
				3	108.9	111.1
BHET Commercial	50	150	3	1	103.1	107.2
				2	103.3	107.1
				3	103.6	107.4
[a]PET residue	50	300	10	1	162	168.2
				2	162.5	168
				3	162.3	167.8

[a]*Reaction Conditions: PET:Cat = 50 (mol/mol), EG:PET = 7 (mol/mol), PS = 0.25 mm, T= 185 °C, t=24 h*

DSC

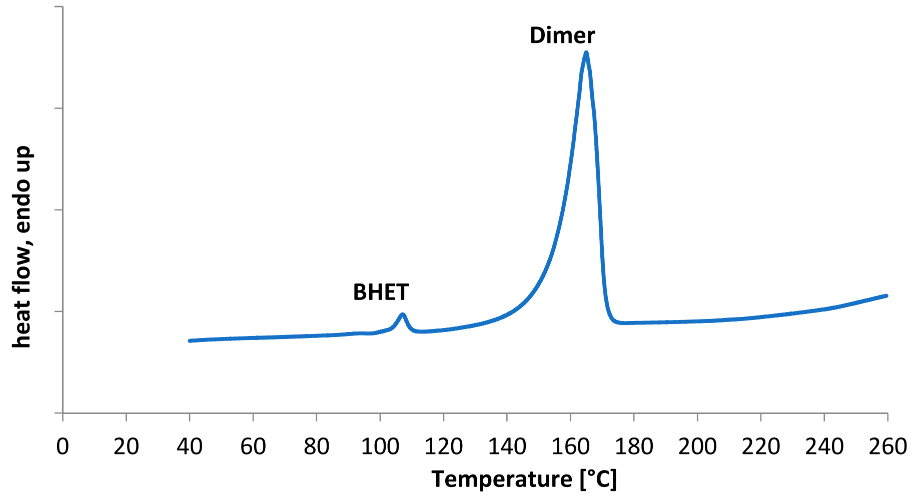

Figure S6: DSC scan of reaction residue

Reaction Conditions: PET:Cat = 50 (mol/mol), EG:PET = 7 (mol/mol), PS = 0.25 mm, T= 185 °C, t=24 h, heating rate (DSC): 10 °C/min

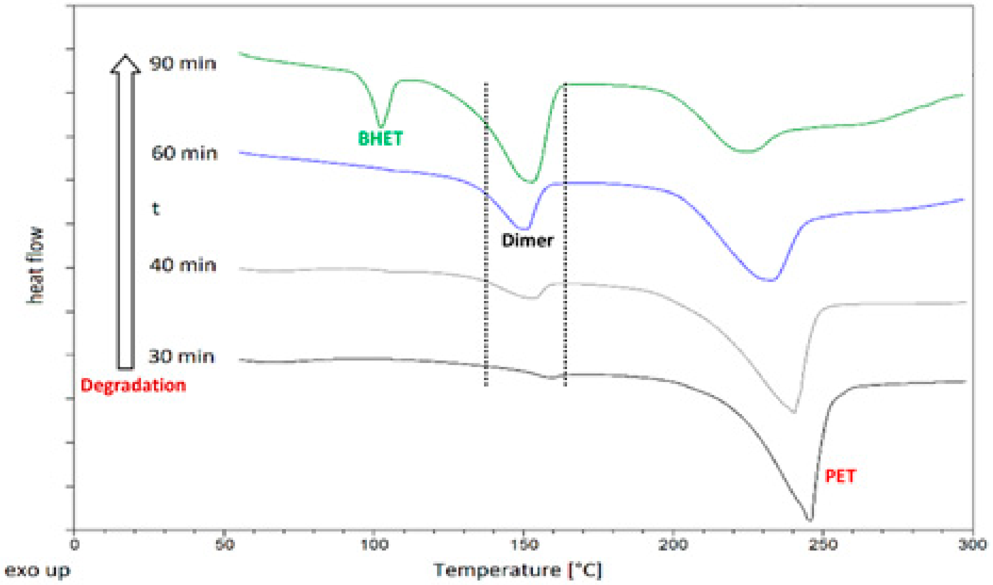

Figure S7: DSC scan of reaction residue with time variations

Reaction Conditions: PET:Cat = 50 (mol/mol), EG:PET = 7 (mol/mol), PS = 0.25 mm, T= 190 °C, t=30-90 min, heating rate (DSC): 10 °C/min

Design of Experiments (DoE)

Table S2: Experimental layout and output responses based on Box-Behnken design

Standard Order	Experimental Run	A(PET: Cat) (mol/mol)	B(EG: PET) (mol/mol)	C(t_R) (h)	D(PS) (mm)	Response (X) (%)
17	1	50.00	5.00	2.00	0.63	70.11
20	2	150.00	5.00	6.00	0.63	70.25
19	3	50.00	5.00	6.00	0.63	73.5
18	4	150.00	5.00	2.00	0.63	39.48
7	5	100.00	5.00	2.00	1.00	41.4
16	6	100.00	7.00	6.00	0.63	83.3
12	7	150.00	5.00	4.00	1.00	64.8
25	8	100.00	5.00	4.00	0.63	70.36
26	9	100.00	5.00	4.00	0.63	69.18
6	10	100.00	5.00	6.00	0.25	68.99
5	11	100.00	5.00	2.00	0.25	53.99
1	12	50.00	3.00	4.00	0.63	53.91
29	13	100.00	5.00	4.00	0.63	71.88
14	14	100.00	7.00	2.00	0.63	54.54
3	15	50.00	7.00	4.00	0.63	85.64
2	16	150.00	3.00	4.00	0.63	52.8
27	17	100.00	5.00	4.00	0.63	63.69
23	18	100.00	3.00	4.00	1.00	56.4
24	19	100.00	7.00	4.00	1.00	78.2
9	20	50.00	5.00	4.00	0.25	79.31
13	21	100.00	3.00	2.00	0.63	37.4
11	22	50.00	5.00	4.00	1.00	69.64
10	23	150.00	5.00	4.00	0.25	57.16
4	24	150.00	7.00	4.00	0.63	63.86
22	25	100.00	7.00	4.00	0.25	86.85
15	26	100.00	3.00	6.00	0.63	56.13
8	27	100.00	5.00	6.00	1.00	69.45
21	28	100.00	3.00	4.00	0.25	54.35
28	29	100.00	5.00	4.00	0.63	67.53

Four different models such as two-factor interaction (2FI), linear, quadratic, and cubic models were used to fit experimental data to generate regression equations. To evaluate the suitability of these models, three different tests were carried out. These tests include the sequential model sum of squares, lack of fit, and model summary statistics and the outcomes are shown in Table 4S. The sequential model sum of squares selects the highest order polynomial where the additional terms are significant and the model is not aliased because the aliased model results in unstable and

inaccurate coefficients and graphs. The lack of fit tests wants the selected model to have insignificant lack-of-fit which means the p-value is greater than 0.05. The model summary statistics focus on the model maximizing the adjusted R^2 and predicted R^2 and a low prediction error sum of squares (PRESS). Based on the aforementioned criteria and the data given in Table 4S, a quadratic model was selected to study the response surface methodology.[1]

Table S3: Fit Summary of the tested models for PET Conversion X

Response: PET Conversion X %						
**Sequential Model Sum of Squares**						
Source	Sum of Squares	dF	Mean Square	F value	p-value prob>F	Remarks
Linear	3582.56	4	895.64	19.44	< 0.0001	
2FI	465.49	6	77.58	2.18	0.0934	
Quadratic	430.15	4	107.54	7.16	0.0023	Suggested
Cubic	70.80	8	8.85	0.38	0.8964	Aliased
Residual	139.42	6	23.24			
"Sequential Model Sum of Squares": Select the highest order polynomial where the additional terms are significant and the model is not aliased.						
**Lack of Fit Tests**						
Linear	1066.44	20	53.32	5.41	0.0564	
2FI	600.95	14	42.93	4.36	0.0828	
Quadratic	170.80	10	17.08	1.73	0.3140	Suggested
Cubic	100.00	2	50.00	5.07	0.0799	Aliased
Pure Error	39.42	4	9.85			
"Lack of Fit Tests": Want the selected model to have insignificant lack-of-fit.						
**Model Summary Statistics**						
Source	Std Dev	R-Squared	Adjusted R-Squared	Predicted R-Squared	PRESS	
Linear	6.79	0.7641	0.7248	0.6441	1668.78	
2FI	5.96	0.8634	0.7875	0.6087	1834.43	
Quadratic	3.88	0.9552	0.9103	0.7770	1045.40	Suggested
Cubic	4.82	0.9703	0.8612	-2.0846	14462.12	Aliased
"Model Summary Statistics": Focus on the model maximizing the "Adjusted R-Squared" and the "Predicted R-Squared".						

Table S4: ANOVA summary and coefficients of the second-order polynomial (quadratic) equation

Actual Factor	Coded factor	Coded Coefficient	Actual Coefficient	Standard Error	Mean Square	F Value	p-value	Significant
Intercept		68.53	17.3737	1.73				
A	C_1	-6.98	-0.29122	1.12	584.64	38.94	< 0.0001	Yes
B	C_2	11.78	16.37	1.12	1666.16	110.96	< 0.0001	Yes
C	C_3	10.39	8.179	1.12	1295.84	86.30	< 0.0001	Yes
D	C_4	1.73	-26.454	1.12	35.91	2.39	0.1443	No
AB	$C_1 \times C_2$	-5.17	-0.05167	1.94	106.81	7.11	0.0184	No
AC	$C_1 \times C_3$	6.84	0.0685	1.94	187.42	12.48	0.0033	No
AD	$C_1 \times C_4$	4.33	0.231	1.94	74.91	4.99	0.0423	No
BC	$C_2 \times C_3$	2.51	0.6269	1.94	25.15	1.67	0.2165	No
BD	$C_2 \times C_4$	-2.67	-3.567	1.94	28.62	1.91	0.1890	No
CD	$C_3 \times C_4$	3.26	4.35	1.94	42.58	2.84	0.1144	No
A^2	C_1^2	-0.10	0.0000403	1.52	0.066	0.00438	0.9482	No
B^2	C_2^2	-2.24	-0.559	1.52	32.42	2.16	0.1638	No
C^2	C_3^2	-7.84	-1.96	1.52	398.76	26.56	0.0001	Yes
D^2	C_4^2	-0.091	-0.645	1.52	0.053	0.003551	0.9533	No
Lack of Fit					17.08	1.73	0.3140	No
Standard deviation = 3.88								

Regression Model in terms of Actual Factors: (significant terms are marked as red)

$$\textbf{PET Conversion, X}\ (\%) = +17.3737 - 0.29122 \times A + 16.37 \times B + 8.179 \times C - 26.454 \times D - 0.05167 \times A \times B + 0.0685 \times A \times C + 0.231 \times A \times D + 0.6269 \times B \times C - 3.567 \times B \times D + 4.35 \times C \times D - 0.0000403 \times A^2 - 0.559 \times B^2 - 1.96 \times C^2 - 0.645 \times D^2 \quad \text{SEq. 1}$$

DoE Model optimization

Table S5: List of the possible optimum set of parameters after DoE optimization

Criteria | Solutions | Graphs

Solutions 1 2 3 4 5 6 7 8 9 10 11 12 13 14 15 16 17 18 19 20 21 22 23 24 25

Constraints

Name	Goal	Lower Limit	Upper Limit	Lower Weight	Upper Weight	Importance
PET:Cat	is in range	50	150	1	1	3
EG:PET	is in range	3	7	1	1	3
tR	is in range	2	6	1	1	3
particle size	minimize	0.25	1	1	1	3
Conversion	maximize	37.4	86.85	1	1	5

Solutions

Number	PET:Cat	EG:PET	tR	particle	Convers	Desirability
1	51.33	5.74	4.82	0.25	87	1.000
2	53.86	6.98	2.11	0.25	87	1.000
3	84.85	6.95	5.64	0.25	88	1.000
4	72.52	6.77	3.51	0.25	88	1.000
5	71.43	6.78	5.95	0.25	88	1.000
6	56.51	6.16	3.78	0.25	90	1.000
7	52.01	5.73	4.42	0.25	88	1.000
8	56.88	6.91	5.19	0.25	95	1.000
9	57.86	6.24	4.50	0.25	91	1.000
10	77.71	6.94	5.00	0.25	91	1.000
11	50.91	6.65	2.27	0.25	88	1.000
12	50.80	6.03	3.34	0.25	89	1.000
13	58.28	6.36	5.50	0.25	89	1.000
14	73.89	6.78	5.21	0.25	90	1.000

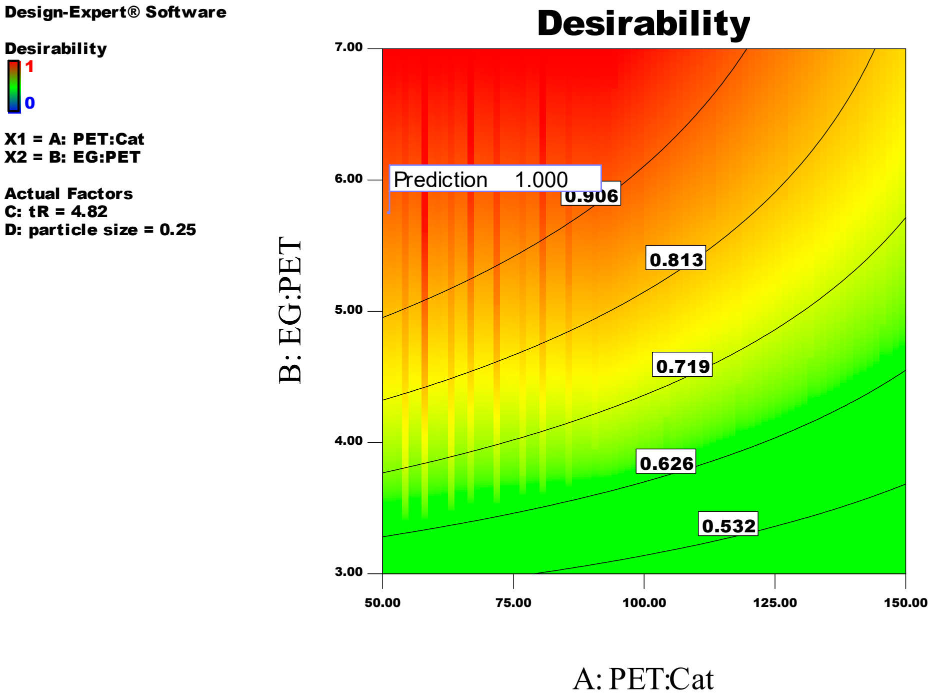

Figure S8: Desirability function of optimum solution 1

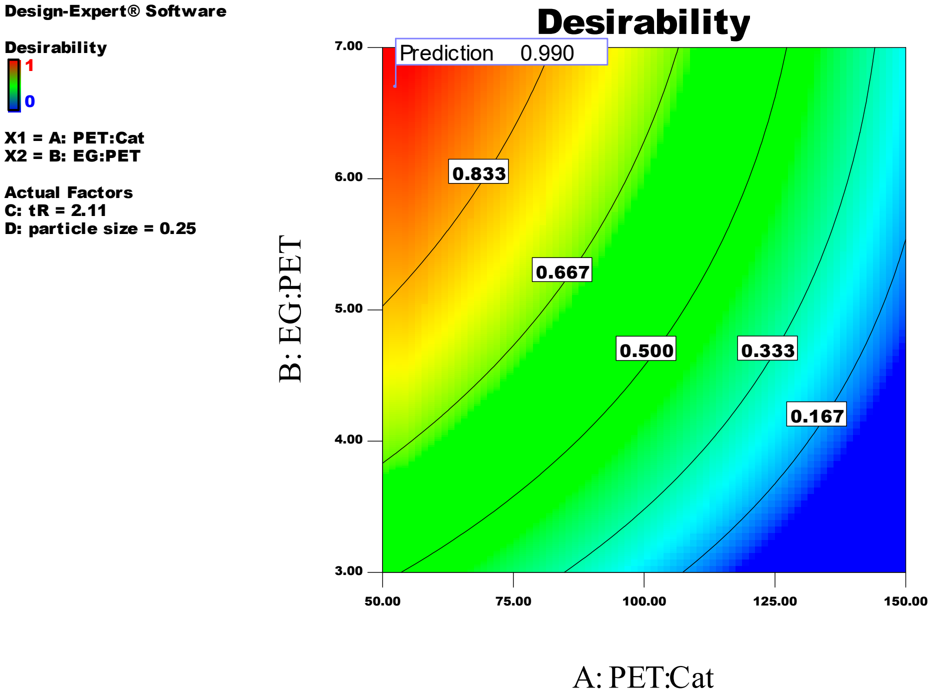

Figure S9: Desirability function of optimum solution 2

Performance comparison of sodium methoxide

Table S6: Cost comparison of sodium methoxide [2–4]

Catalyst	Product No.	Purity (%)	Cost (€)		PET Conversion[b] (%)	TON (mol/mol)	TOF^{c}_{20} (h^{-1})
			100 g container	Per 1 mol of catalyst[a]			
$Zn(OAc)_2$	383317	>99.9	304	557	86	40	314
$Co(OAc)_2$	399973	>99.9	217[d]	3840[d]	82	40	54
MeONa	164992	95	26.90	15	78	39	58

[a]Based on 100 g container, [b]Reaction Conditions: T = 190 °C, time = 0.5-1.5 h, PET: Cat = 50 (mol/mol), EG: PET = 7 (mol/mol), PS = 0.25 mm, [c]TOF calculated for 20% PET conversion, [d] Based on 10 g container

List of Abbreviations

Abbreviation	Meaning
BHET	bis(2-hydroxyethyl)terephthalate
$Co(OAc)_2$	Cobalt acetate (anhydrous)
DoE	Design of Experiments
DSC	Differential Scanning Calorimetry
EG	Ethylene glycol
GC-MS	Gas Chromatography-Mass Spectroscopy
MeONa	Sodium methoxide
NMR	Nuclear Magnetic Resonance
PET	Poly(ethylene terephthalate)
RSM	Response surface methodology
TON	Turn over number
TOF	Turn over frequency
$Zn(OAc)_2$	Zinc acetate (anhydrous)

References

[1] A.G. Rana, M. Minceva, Analysis of Photocatalytic Degradation of Phenol with Exfoliated Graphitic Carbon Nitride and Light-Emitting Diodes Using Response Surface Methodology, Catalysts 11 (2021) 898. https://doi.org/10.3390/catal11080898.

[2] Sodium methoxide | Sigma-Aldrich, 2023.000Z. https://www.sigmaaldrich.com/DE/en/search/sodium-

methoxide?focus=products&page=1&perpage=30&sort=relevance&term=sodium%20methoxide&type=product (accessed 19 January 2023.413Z).
[3] Cobalt acetate | Sigma-Aldrich, 2023.000Z. https://www.sigmaaldrich.com/DE/en/search/cobalt-acetate?focus=products&page=1&perpage=30&sort=relevance&term=cobalt%20acetate&type=product (accessed 19 January 2023.013Z).
[4] Zinc acetate | Sigma-Aldrich, 2023.000Z. https://www.sigmaaldrich.com/DE/en/search/zinc-acetate?focus=products&page=1&perpage=30&sort=relevance&term=zinc%20acetate&type=product (accessed 19 January 2023.079Z).
